# Supplementary material for: Augmenter of liver regeneration regulates cellular iron homeostasis by modulating mitochondrial transport of ATP-binding cassette B8
Source: eLife. 2021 Apr 9;10:e65158. doi: 10.7554/eLife.65158 (PMC8055271; doi:10.7554/eLife.65158)
Supplement: Supplementary file 1. — The protocol for qRT-PCR were described in 'Materials and methods' section. [file elife-65158-supp1.docx]

Supplemental table 1 Primer Sequences

| **Human Genes** | **Forward Primer** | **Reverse Primer** |
| --- | --- | --- |
| 18s | AGTCCCTGCCCTTTGTACACA | CGATCCGAGGGCCTCACTA |
| β-actin | AGGATGCAGAAGGAGATCACTG | GGGTGTAACGCAACTAAGTCATAG |
| HPRT | AATTCTTTGCTGACCTGCTG | CTTTTATGTCCCCTGTTGACTG |
| GAPDH | TAGAGGGGTGATGTGGGGAG | AGTGATGGCATGGACTGTGG |
| TFRC | AAAATCCGGTGTAGGCACAG | CACCAACCGATCCAAAGTCT |
| ALR | TAAGTTTTACCCCTGTGAGG | CTGCACAGCCTTTTTCTTAG |
| ABCB7 | AACTCGGCGCCTTGGGAACC | TCAGTGGCCATACCTGGAGAGCC |
| ABCB8 | ACCAGCTTCCCCGAGGGCTAC | GCGCGCTGGTAGCTTCATCCA |
| **Mouse Genes** | **Forward Primer** | **Reverse Primer** |
| 18s | AGTCCCTGCCCTTTGTACACA | CGATCCGAGGGCCTCACTA |
| β-actin | CCGTGAAAAGATGACCCAGAT | GTACATGGCTGGGGTGTTG |
| HPRT | CTGGAAAGAATGTCTTGATTGTTG | TGCATTGTTTTACCAGTGTCAA |
| β2-microglobulin | TGGTCTTTCTGGTGCTTGTC | CAGTTCAGTATGTTCGGCTTC |
| Tfrc | GCATTGCGGACTGTAGAGG | GCTTGATCCATCATTCTCAGC |
| ALR | CCCTGCGAGGAATGTGCGGAA | TCACCTCATTGTGCAGGCGGC |
| IRP1 | TTTCAACAGAAGGGCAGACA | TTTCTTTCAAACTCCAGGTCTTG |
